# Impaired cardiac glycolysis and glycogen depletion are linked to poor myocardial outcomes in juvenile male swine with metabolic syndrome and ischemia

**DOI:** 10.14814/phy2.15742

**Published:** 2023-08-03

**Authors:** Mark Broadwin, Dwight D. Harris, Sharif A. Sabe, Elif Sengun, Amber J. Sylvestre, Boian S. Alexandrov, Frank W. Selke, Anny Usheva

**Affiliations:** ^1^ Division of Cardiothoracic Surgery, Department of Surgery Warren Alpert Medical School of Brown University Providence Rhode Island USA; ^2^ Division of Cardiology, Department of Medicine Warren Alpert Medical School of Brown University Providence Rhode Island USA; ^3^ Los Alamos National Laboratory Los Alamos New Mexico USA

**Keywords:** myocardial metabolomics, nonnegative matrix factorization, proteomics, swine model of metabolic syndrome

## Abstract

Obesity continues to rise in the juveniles and obese children are more likely to develop metabolic syndrome (MetS) and related cardiovascular disease. Unfortunately, effective prevention and long‐term treatment options remain limited. We determined the juvenile cardiac response to MetS in a swine model. Juvenile male swine were fed either an obesogenic diet, to induce MetS, or a lean diet, as a control (LD). Myocardial ischemia was induced with surgically placed ameroid constrictor on the left circumflex artery. Physiological data were recorded and at 22 weeks of age the animals underwent a terminal harvest procedure and myocardial tissue was extracted for total metabolic and proteomic LC/MS–MS, RNA‐seq analysis, and data underwent nonnegative matrix factorization for metabolic signatures. Significantly altered in MetS versus. LD were the glycolysis‐related metabolites and enzymes. In MetS compared with LD Glycogen synthase 1 (GYS1)‐glycogen phosphorylases (PYGM/PYGL) expression disbalance resulted in a loss of myocardial glycogen. Our findings are consistent with the concept that transcriptionally driven myocardial changes in glycogen and glucose metabolism‐related enzymes lead to a deficiency of their metabolite products in MetS. This abnormal energy metabolism provides insight into the pathogenesis of the juvenile heart in MetS. This study reveals that MetS and ischemia diminishes ATP availability in the myocardium via altering the glucose‐G6P‐pyruvate axis at the level of metabolites and gene expression of related enzymes. The observed severe glycogen depletion in MetS coincides with disbalance in expression of GYS1 and both PYGM and PYGL. This altered energy substrate metabolism is a potential target of pharmacological agents for improving juvenile myocardial function in MetS and ischemia.

## 1 INTRODUCTION

Childhood obesity rates have increased significantly, with 35% of children aged 10–11 now diagnosed with obesity (Abaci et al., 1999; Cote et al., 2013; Grundy et al., 2002). Obese children are more likely to develop metabolic syndrome (MetS) and concurrent cardiovascular abnormalities, suggesting that there is a predisposition to short‐term cardiovascular injury and increased risk of long‐term cardiovascular disease (CVD) (Cote et al., 2013).

MetS is a complex disorder defined by the presence of at least three of the following conditions: increased waist circumference, increased low‐density lipoproteins (LDL), hypertriglyceridemia, hyperglycemia, and hypertension. While previous research has identified a number of signaling pathways, individual proteins, and metabolites involved in the myocardial response to MetS, these discoveries are limited to correlations and mostly reflect reactive processes rather than causative factors. Although MetS is strongly associated with the development of coronary artery disease (CAD), the true nature of this relationship is not yet fully understood. Unfortunately, effective prevention and long‐term treatment options for CVD are limited. As a result, the risk of long‐term cardiovascular damage remains high in obese children.

Analyses of blood or blood components are unlikely to capture all of the metabolic alterations that the myocardium undergoes in MetS due to the myocardium's specific gene expression pattern and metabolic characteristics (Karimi et al., 2020; Ziegler et al., 2021). The difficulty in obtaining human cardiac tissue, as well as the significant differences in MetS between humans and small animal models, complicate the identification of myocardium‐specific risk factors in MetS (Abel, 2021; Ahluwalia et al., 2015).

To better understand MetS and the risks of cardiac illness observed in the clinical setting, we investigated MetS using a juvenile swine model that closely mirrors the diagnostic criteria for MetS and risks of cardiac illness seen in humans (Gerrity et al., 2001). When compared to the lean diet (LD) controls, the high‐calorie, high‐fat obesogenic diet produced the classic features of MetS: hyperglycemia, significant increase in triglycerides, increased plasma LDL, increased total cholesterol, weight gain, and higher systolic and diastolic blood pressure (Bugger et al., 2022; Neeb et al., 2010).

A significant contributor to ischemic heart disease, atherosclerosis is a chronic process that has been shown to start years before the onset of ischemic insult. In fact, it is well known that this accidental process starts in childhood. In order to understand how this disease develops rather than just the result of a chronic pathology, we decided to study chronic ischemia in a juvenile model. The specific goal of this study is to determine the juvenile cardiac metabolomic response to MetS in a swine model of chronic myocardial ischemia. We hypothesized that MetS and chronic ischemia would impair energy supply in the juvenile myocardium through altered metabolism and protein expression.

## 2 METHODS

### 2.1 Animal model

Intact (non‐castrated) male Ossabaw swine aged 4 weeks were fed either an obesogenic diet (OD, *n* = 6), which we have previously shown induces effects analogous to MetS in humans (hyperlipidemia and impaired glucose tolerance), or a control normoglycemic lean diet (LD, *n* = 6) for 11 weeks, as previously described (Robich et al., 2010). The high‐fat obesogenic diet group swine were given daily feeds consisting of 500 g, 2248 kcal/d obesogenic diet composed of 4% cholesterol, 17.2% coconut oil, 2.3% corn oil, 1.5% sodium cholate, and 75% regular chow (Sinclair Research). The LD group received 1824 kcal/d regular chow (Sinclair Research). To induce chronic myocardial ischemia, an ameroid constrictor was placed on the left circumflex femoral artery at 15 weeks of age. This caused gradual stenosis of the artery over 10–20 days. The corresponding dietary regimens were continued for 7–8 weeks. At 22 weeks of age, the animals underwent a terminal harvest procedure where coronary perfusion and overall hemodynamic function were assessed. Left ventricular tissue was divided into ten small segments according to territorial distribution and classified as ischemic (distribution of the left circumflex artery, LCx) and were rapidly frozen in liquid nitrogen. At the time of harvest, the 22‐week‐old Ossabaw swine would be the approximate age of a 14–16 year old in human terms. At age 4 weeks diet an obesogenic diet is initiated and continued throughout the remainder of the experiment. At 15 weeks of age swine underwent ameroid placement. At 22 weeks of age pigs underwent harvest procedure. The model in use enables the analysis of well‐perfused cardiac tissue segments as controls. Although they share a common ancestor, this model has been extensively studied and is frequently mentioned in the literature. To find and confirm well‐perfused non‐ischemic segments, gold microsphere perfusion data is used. Animal care was conducted per Principles of Laboratory Animal Care guidelines by the National Society for Medical Research and the Guide for the Care and Use of Laboratory Animals (NIH publication number 5377–3, 1996). The study was conducted in compliance with the ARRIVE guidelines.

### 2.2 Liquid chromatography with tandem mass spectroscopy (LC/MS–MS)‐based metabolomics

Metabolites were extracted from 100 mg flash‐frozen tissue or 0.2 mL blood with 1 mL of ice‐cold 80% (v/v) methanol and 0.6 mL acetonitrile. Samples were analyzed using a 5500 QTRAP hybrid triple quadrupole mass spectrometer (AB/SCIEX) coupled to a Prominence UFLC HPLC system (Shimadzu) with SRM, as previously reported (Karimi et al., 2020; Yuan et al., 2012; Ziegler et al., 2021). LC/MS–MS was conducted for the individuals pig samples. MetaboAnalyst 5.0 was used to identify known pathway participation. All analyses are conducted in agreement with the biosafety regulations at the Rhode Island Hospital, Providence, RI. All research was conducted in accordance with the ethical standards and principles at the Warrne Alpert School of Medicine at Brown University and Rhode Island Hospital.

### 2.3 Nonnegative matrix factorization (NMF)

NMF was applied to search for metabolic signatures in the LC/MS–MS data set of 305 metabolites in a total of 12 swine (obesogenic diet *n* = 6, LD *n* = 6). Hierarchical clustering was performed (Cichocki et al., 2009; Ziegler et al., 2021). All simulations were run on Linux clusters at the Los Alamos National Laboratory.

### 2.4 LC/MS–MS‐based proteomics and RNA‐seq

As previously described 100 mg of left ventricular myocardial tissue was lysed in lysis buffer (8 M urea, 1 mM sodium orthovanadate, 20 mM HEPES, 2.5 mM sodium pyrophosphate, 1 mM β‐glycerophosphate, pH 8.0, 20 min, 4°C). 200 μg of protein per sample was subjected for trypsin digestion. LC/MS–MS was conducted on a fully automated proteomic technology platform that includes an Agilent 1200 Series Quaternary HPLC system (Agilent Technologies) connected to a Q Exactive Plus mass spectrometer (Thermo Fisher Scientific). (Agilent Technologies) and Q Exactive Plus mass spectrometer (Thermo Fisher Scientific). The MS/MS spectra were acquired at a resolution of 17,500, with a targeted value of 2 × 104 ions or maximum integration time of 200 ms. The ion selection abundance threshold was set at 8.0 × 102 with charge state exclusion of unassigned and *z* = 1, or 6–8 ions, and dynamic exclusion time of 30 s. Peptide spectrum matching of MS/MS spectra of each file was searched against the NCBI *Sus scrofa* database (Taxon ID: 9823, downloaded on 11/21/2019) using the Sequest algorithm within Proteome Discoverer v 2.3 software (Thermo Fisher Scientific). The Sequest database search was performed with the following parameters: trypsin enzyme cleavage specificity, two possible missed cleavages, 10 ppm mass tolerance for precursor ions. Peptide assignments from the database search were filtered down to a 1% FDR. The relative label‐free quantitative and comparative among the samples were performed using the Minora algorithm and the adjoining bioinformatics tools of the Proteome Discoverer 2.3 software. To select proteins that show a statistically significant change in abundance between two groups, a threshold of 1.5‐fold change with *p*‐value (0.05) were selected (Karimi et al., 2020; Ziegler et al., 2021). Total mRNA was used for RNA‐seq at Next Generation Sequencing (Azenta US, Inc).

### 2.5 Western blot

Sample lysates were prepared by homogenizing tissue samples with RIPA buffer (Boston BioProducts). Following lysate preparation, Western blots were conducted (50 μg of protein/lane), as previously reported, using Glycogenin‐1 antibody (E‐11, sc‐271,109; Santa Cruz Biotechnology) and goat‐anti‐Rabbit IgG, Alexa Fluor 594 (Thermo Fisher Scientific; Cat # A‐11012) (Robich et al., 2010). Transferred membranes were stained with Ponceau S as an internal control to confirm equal protein loading and transfer (Figure S3).

### 2.6 Glycogen content

Periodic acid Schiff (PAS) staining was conducted on deparaffinized and rehydrated to water tissue sections, as suggested by the kit supplier (VWR, 75877–056, 75,877–058). Following PAS staining, tissue sections underwent α‐amylase treatment as a control.

### 2.7 Data analysis

Data analysis was performed using Microsoft Excel and GraphPad Prism 7 software (GraphPad Software, Inc, San Diego, CA). For statistical analysis unpaired two‐tail Student's *t*‐test was used to compare differences between 2 datasets. Data are presented as means ± SD. In all cases, *p* < 0.05 was considered to be a statistically significant difference. Functional biological data analysis was conducted with the list of genes that were differentially expressed (*p* < 0.05) in response to diet using the g:Profiler approach (Raudvere et al., 2019).

## 3 RESULTS

### 3.1 Obesogenic diet resulted in MetS in our juvenile swine model

**FIGURE 1:**
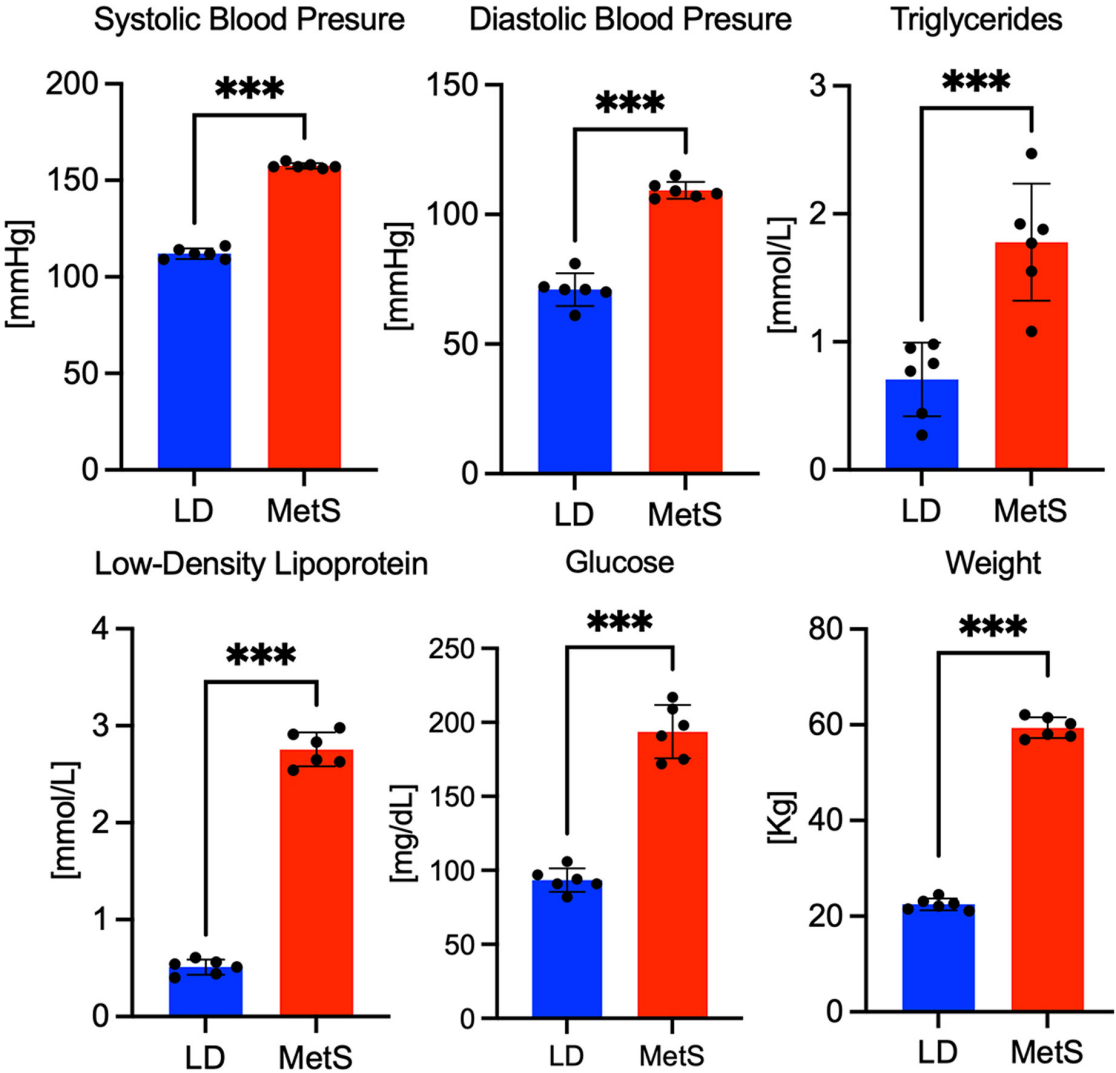
Juvenile swine fed an obesogenic diet developed key physiological components of MetS. The bar graphs display diet‐related differences in glucose (*n* = 12), low‐density lipoproteins (*n* = 12), triglycerides (*n* = 12), systolic blood pressure (*n* = 12), diastolic blood pressure (*n* = 12), and weight (*n* = 12), as shown above the bars. Blue bars represent LD; red bars represent MetS. Data is presented as means ± SD; ***p < 0.001, 2‐tailed Student's *t*‐test.

After 12 weeks of a high‐fat, high‐calorie diet, all swine in the MetS obesogenic diet group (MetS) exhibited higher fasting plasma glucose (*n* = 6, 193 ± 18 mg/dL vs. 94 ± 12 mg/dL, *p* = 0.02), triglycerides (1.78 ± 0.7 mmol/L vs. 0.71 ± 0.27 mmol/L), plasma LDL (2.76 ± 0.22 mmol/L vs. 0.51 ± 0.11 mmol/L, *p* = 0.02), total cholesterol (6.2 mmol/L ± 0.5 vs. 1.4 ± 0.4 mmol/L, *p* = 0.007), increased weight gain (59 ± 3.1 kg vs. 22 ± 2.5 kg), and systolic (157 ± 3 mmHg vs. 112 ± 4 mmHg, *p* = 0.005) and diastolic (109 ± 6 mmHg vs. 71 ± 10 mmHg, *p* = 0.003) blood pressure versus. swine in the LD group (Figure 1).

Obesity, high fasting blood sugar, high LDL, elevated triglyceride levels, and high blood pressure are the five MetS diagnostic criteria observed in juvenile pigs on the obesogenic diet, indicating that the juvenile pigs developed MetS in response to this diet.

### 3.2 Metabolomic analyses uncovered diet‐related alterations in the abundance of several myocardial polar metabolites

**FIGURE 2:**
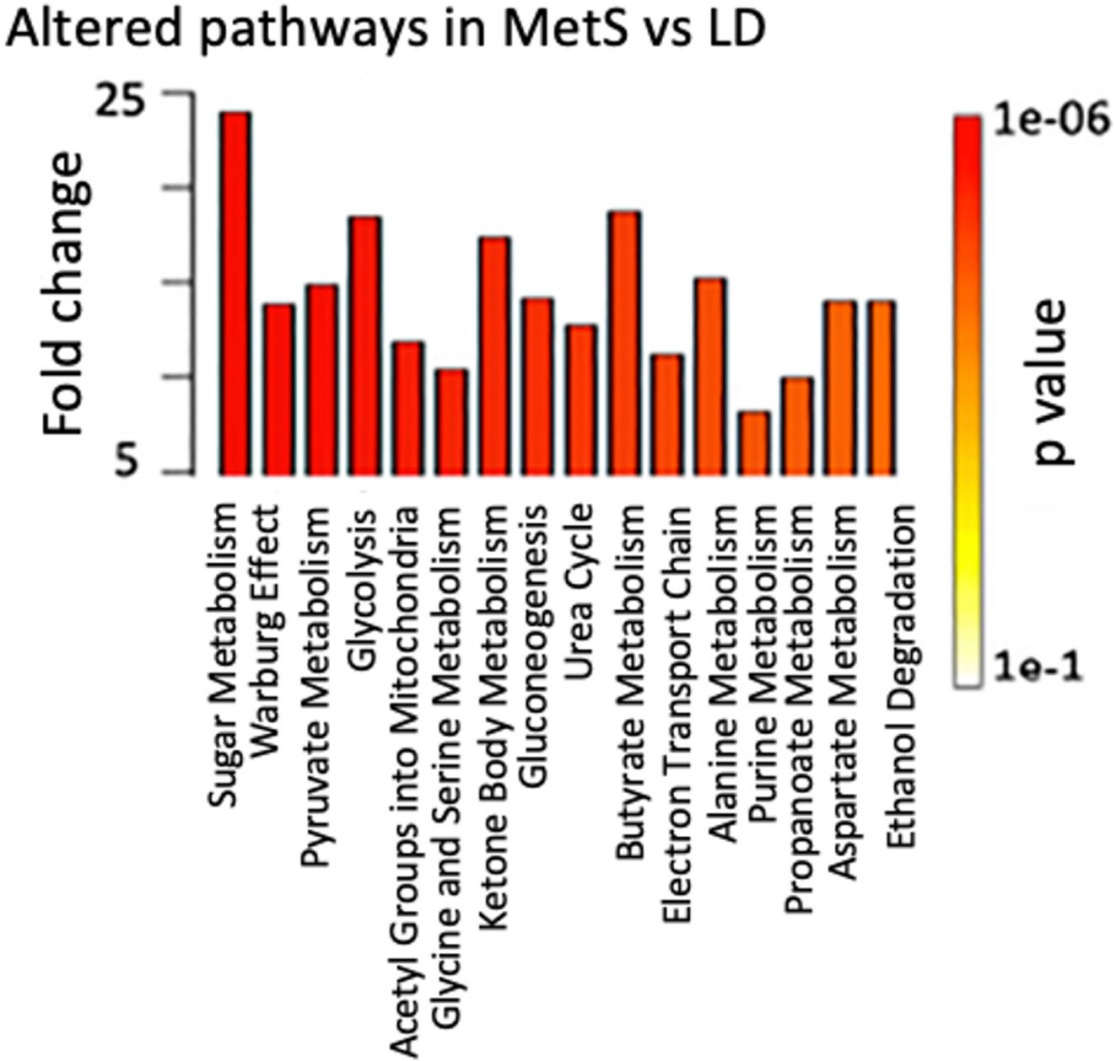
NMF selected overrepresented metabolomic changes in the juvenile MetS heart. The polar metabolites were extracted from left ventricular cardiac tissue (50 mg). LC/MS–MS was applied to identify and semi‐quantitatively measure the content of individual polar metabolites from swine (MetS *n* = 6, LD *n* = 6). The bar diagram shows overrepresented pathways in MetS versus. LD. Their p values are shown with the colored bar on right.

To identify metabolite signatures from juvenile MetS and LD ischemic myocardial tissues, we used targeted liquid chromatography combined with tandem mass spectrometry (LC/MS–MS) for polar metabolites. For each myocardial sample, 283 metabolites were identified and quantitatively compared. Six MetS and six LD samples were analyzed in a single experiment to prevent batch effects. The obtained mass spec data are challenging to statistically analyze since there are 283 metabolites and less replicates (12 pigs). Because of this, we used an unsupervised learning method based on NMF to compare variations in the metabolite profiles in MetS versus. LD based on the specific proportion of the 283 metabolites in each swine (Cichocki et al., 2009; Ziegler et al., 2021). The differentially represented processes underlying the metabolomics signature in the MetS swine (Figure 2) are related to sugar metabolism (*p* = 4e‐5), Warburg effect (*p* = 3.4e‐5), glycolysis, and butyrate metabolism (*p* = 1.1e‐4). Also overrepresented in MetS are processes related to the urea cycle (*p* = 0.004). NMF identified 27 metabolites as being overrepresented in LD versus. MetS based on the weight of the metabolite with a higher probability in LD than in MetS (Table S1).

RNA‐Seq was performed on the same swine tissues and the results were correlated with the expression of genes involved in sugar and glycolysis metabolism. To validate the RNA‐Seq results at the protein level, mass spectroscopy was performed on three LD and three MetS parallel samples. From the RNA‐Seq and proteomics LC–MS/MS data, genes and proteins that showed a threshold of a 1.5‐fold change were selected for analysis. To perform pathway‐related and biological process‐related analyses, we used g:Profiler (Figure S1). The g:Profiler revealed the following major classifications of the overrepresented biological pathways that were altered in MetS swine: small molecule binding (*p*_adj_ = 1.44e‐5), nucleotide binding (*p*_adj_ = 1.07e‐4), glucose binding (*p*_adj_ = 2.97e‐4), carbohydrate derivative binding (*p*_adj_ = 1.44e‐3), and glycogen phosphorylase activity (*p*_adj_ = 2.74e‐3). We confirmed specific transcriptomic changes for individual swine using data from parallel LC–MS/MS proteomics and targeted LC–MS/MS metabolomics.

### 3.3 MetS alters the availability of glycolysis‐related metabolites

**FIGURE 3:**
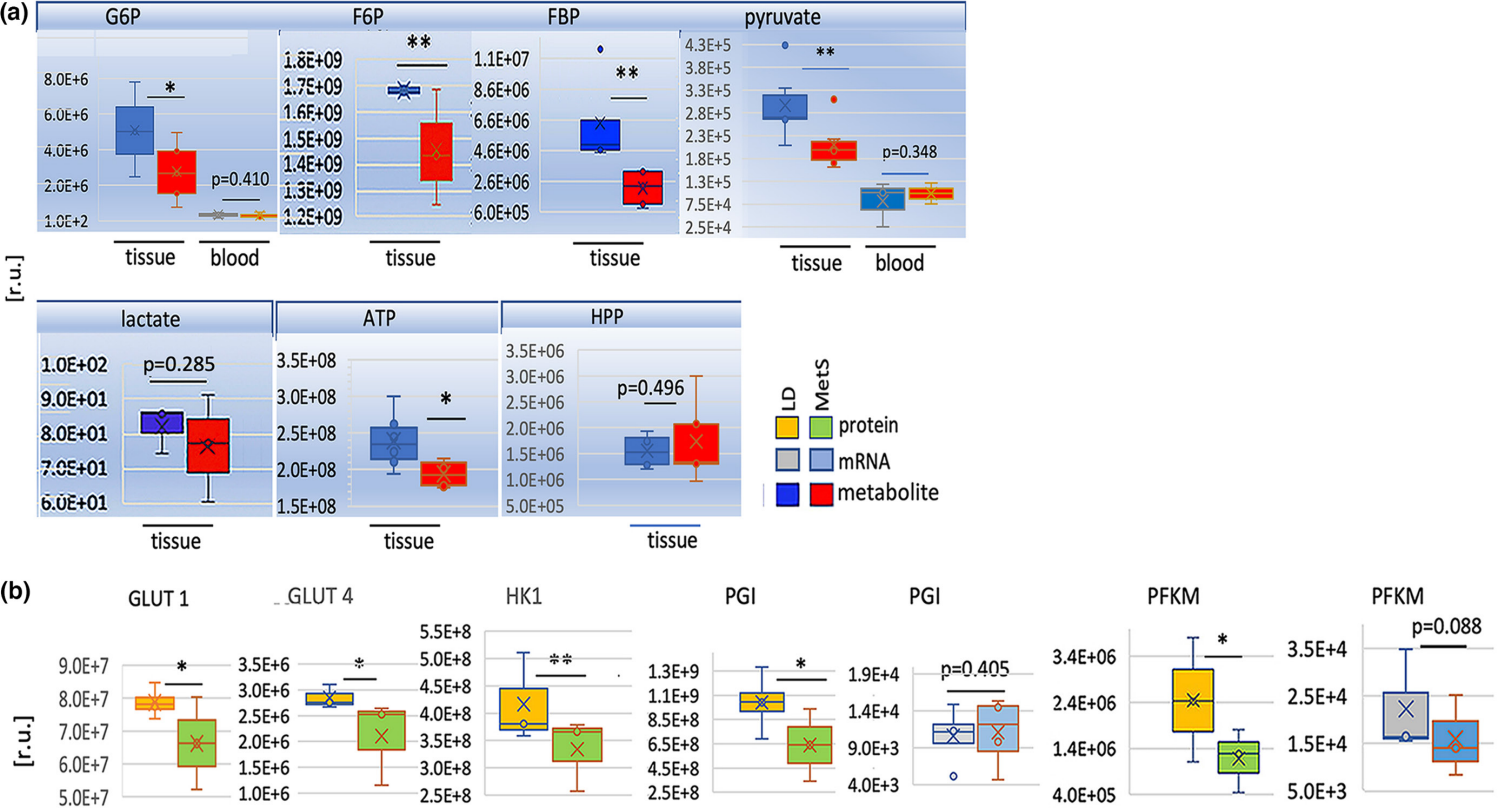
The availability of metabolites related to glycolysis was significantly reduced in the juvenile myocardium in response to MetS. (a) Targeted polar metabolites were identified by LC/MS–MS in extracts from LD and MetS swine myocardial tissues. Myocardial metabolite libraries from LD and MetS pigs were compared for relative content of metabolites that changed in response to MetS. Metabolites from the glycolysis pathway that were significantly altered in response to diet include G6P, F6P, FBP, pyruvate, lactate, and ATP, which are shown above each graph. Metabolite availability was compared in tissue and in blood, as shown below each graph. HPP content did not significantly alter in tissue and was used as a control for the equal loading of metabolites. Blue bars represent LD metabolites; red bars represent MetS metabolites. Values are expressed in relative units (r.u.) and are presented as means ± SD, *p ≤ 0.050, **p < 0.050; 2‐tailed Student's *t*‐test. (b) MetS versus. LD changes in mRNA and/or protein levels are shown for genes with essential functions in glycolysis identified by RNA‐seq and mass spectroscopy: glucose transporters GLUT1 and, GLUT4; hexokinase 1 (HK1), glucose‐6‐phosphate isomerase (PGI), and the muscle isoform of phosphofructokinase (PFKM). The RNA‐seq data is shown in gray for LD (*n* = 4) and blue for MetS (*n* = 4); the mass spec protein data is shown in orange for LD and green for MetS. Gene identities and p values are shown on top of each bar diagram. Values are expressed in relative units (r.u.) on the vertical axes and are presented as means ± SD; *p = 0.050, **p < 0.05; Student's *t*‐test.

The quantitative LC/MS–MS data (Figure 3a) derived from six MetS and six LD independent runs show a significant decrease in MetS versus LD of glucose‐6‐phosphate (G6P, *n* = 12, *p* = 0.001) and fructose‐6‐phosphate (F6P, *n* = 12, *p* = 0.01), both of which are glycolysis entry metabolites. Less abundant in MetS is fructose 1,6‐bisphosphate (FBP, *n* = 12, *p* = 0.01) from the ATP reproductive glycolysis step. The juvenile MetS myocardium displayed a significantly diminished presence of pyruvate (*n* = 12, *p* = 0.004), which represents glycolysis completion. Lactate availability was reduced to a lesser extent (*n* = 12, *p* = 0.285). Hydroxyphenylpyruvate HPP (*n* = 12, *p* = 0.496) and N‐acetyl‐L‐alanine (*n* = 12, *p* = 0.736) were used as a control for the equal loading content of metabolites as the change in tissue content are not significant (Figure 3; Figure S2b).

Concomitantly, the corresponding blood samples contain significantly less G6P (*n* = 12, *p* = 0.410) and pyruvate (*n* = 12, *p* = 0.348) in MetS swine compared to LD swine with no diet‐related difference (Figure 3a). D‐glyceraldehyde‐3‐phosphate and ascorbic acid levels in the blood are, however, significantly higher than in the tissues, and diet has no effect on this difference (S2C). In LD, however, there is more blood ascorbic acid than in MetS (S2C). D‐sedohephulose‐1‐7‐phosphate was used as an equal loading content in both tissue and blood (S2D).

Although the exact flux of myocardial pyruvate and the pathway‐related metabolites remains to be determined, these data support the hypothesis that in MetS and hyperglycemia, glycolysis in the juvenile myocardium is severely diminished. Blood metabolite concentrations have no relationship with myocardial tissue metabolite concentrations or dietary responses.

Given the imbalance of intermediate and glycolysis entry metabolites, MetS myocardium was expected to show differences in the gene expression of enzymes involved in the glycolysis cascade. Therefore, the mRNA content of the genes involved in the glycolysis processes in MetS and LD were compared (Figure 3b). A portion of the mRNA findings were recapitulated at the protein level with LC/MS–MS.

The RNA‐Seq‐based transcriptomics and LC/MS–MS‐based proteomics data derived from sequencing left ventricular myocardial total mRNA libraries and tissue extracts from four swine of each group showed a significantly altered presence of enzymes with functions in glycolysis. Significantly decreased in MetS versus. LD is protein content of the basal glucose transporter 1 (GLUT1, *p* = 0.041, *n* = 8), the insulin‐responsive glucose transporter 4 (GLUT4, *p* = 0.035, *n* = 8), and the mitochondrial hexokinase 1 (HK1, *p* = 0.002, *n* = 8), which catalyzes the rate‐limiting and first obligatory step of glucose metabolism. The protein level of the glucose‐6‐phosphate isomerase (PGI, *p* = 0.038, *n* = 8), which catalyzes the reversible isomerization of G6P to F6P, was also significantly reduced. It is noteworthy that MetS had no effect on the PGI mRNA (Figure 3b). The expression of the phosphofructokinase (PFKM protein, *p* = 0.03, mRNA *p* = 0.10, *n* = 8), a key regulatory enzyme of glycolysis that catalyzes the phosphorylation of F6P to FBP (Figure 3a), was diminished at both the mRNA and protein level.

tRNA (guanine‐N(7)‐)‐methyltransferase, METTL1 (mRNA, *p* = 0.4), P2Y purinoceptor 6, P2RY6 (mRNA, *p* = 0.892), P2Y purinoceptor 12, P2RY12 (mRA, *p* = 0.664) and desmin (mRNA, *p* = 0.592, protein, *p* = 0.791) expression levels did not show diet‐related mRNA or protein content differences. Desmin was used as internal controls for the above observations and have traditionally been used as such in cardiac literature (Figure S2).

These findings are consistent across all independent studies that used both myocardial tissue mRNA libraries and parallel protein lysates from four MetS and four LD pigs. They show that myocardial ischemia and MetS specifically and significantly suppress key glycolysis enzymes and related metabolites.

### 3.4 MetS and ischemia correlate with dramatically diminished myocardial glycogen deposition

**FIGURE 4:**
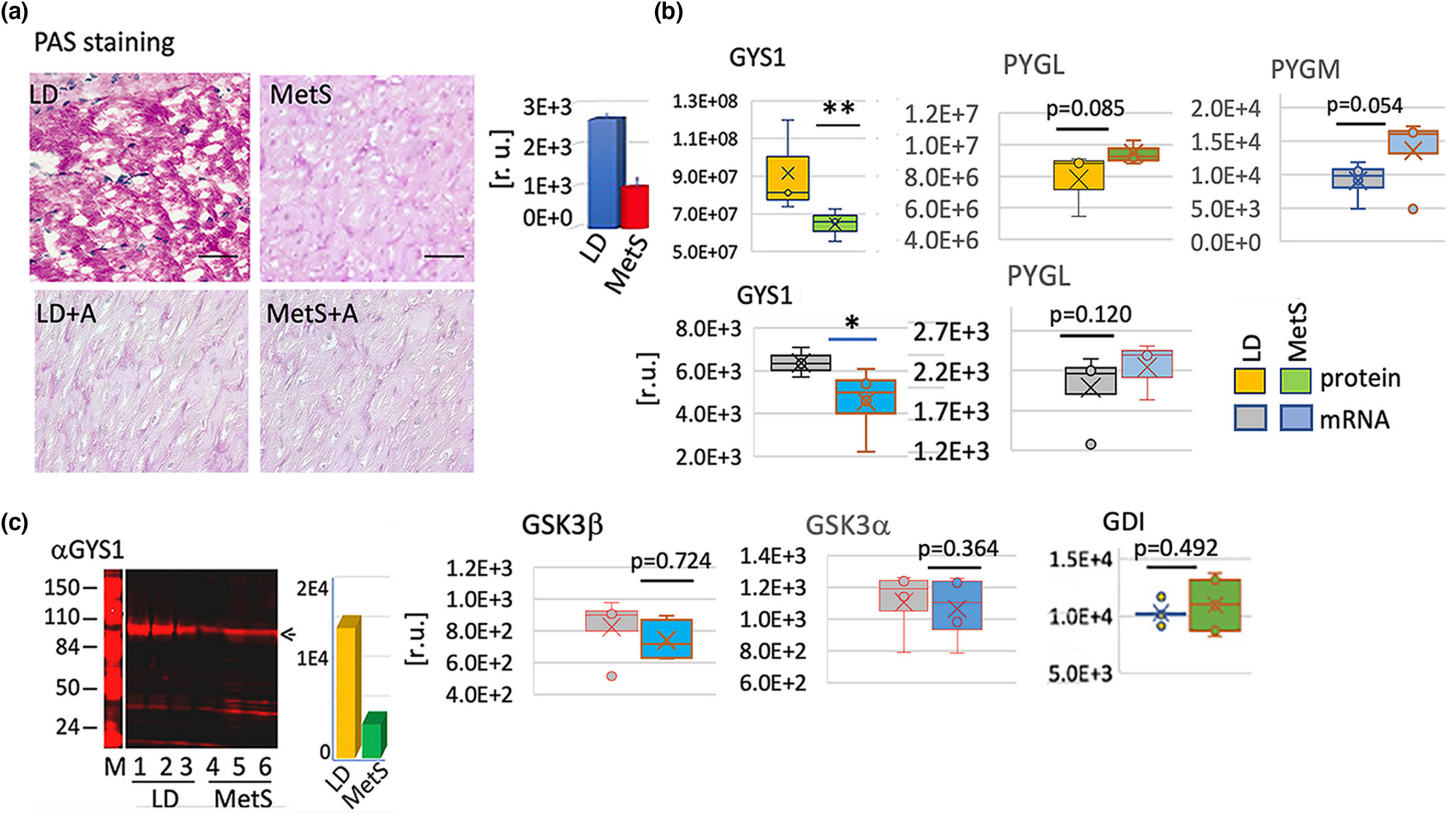
MetS stimulated glycogen deficiency in the juvenile myocardium. (a) PAS staining for glycogen (lilac) of tissue sections from MetS and LD; α‐amylase treatment followed by PAS (MetS+A, LD + A). The bars on the right present the average expressed in relative units (pixels, after subtraction of the signal following α‐amylase treatment). (b) MetS versus. LD changes in expression at the mRNA and/or protein levels are shown for genes with essential functions in glycogen content identified by RNA‐seq and/or mass spectroscopy: GYS1, PYGL, PYGM, GSK3α, GSK3β, and GDI. The RNA‐seq data is shown in gray for LD (*n* = 4) and blue for MetS (*n* = 4); the mass spec protein data is shown in orange for LD and green for MetS. The gene identity and *p* values are shown on top of the bar diagrams. Values are expressed in relative units (r.u.) on the vertical axes and are presented as means ± SD; *p < 0.05, **p < 0.005 (Student's *t*‐test). (**c**) Western blot results showed the amount of GYS1 present in tissue lysates (protein 50 μg/lane) from three representative LD (1, 2, 3) and MetS (4, 5, 6) pigs. Lane M displays the migration of the molecular marker with molecular masses shown on the left. The bar graph on the right displays the fold change of GYS1 in MetS (n = 3) versus LD (*n* = 3) lysates in relative units (r.u.). The calculations were conducted in NIH Image 1.31.

Besides glucose, endogenous glycogen can be catabolized via glycolysis after enzymatic conversion to a glycolytic intermediate. The periodic acid Schiff (PAS) staining of tissue sections revealed that glycogen was significantly less abundant in MetS versus LD. (Figure 4a) and that amounts of glycogen are reproducibly lower in MetS versus. LD. Moreover, the MetS myocardium showed a significant decrease in the content of glycogen synthase 1 (GYS1), which participates in the synthesis of muscle glycogen (Figure 4b) (Browner et al., 1989). RNA‐seq and protein mass spec data demonstrated significantly diminished levels of GYS1 mRNA and GYS1 protein in MetS versus LD (protein *p* = 0.001, mRNA *p* = 0.03, *n* = 8). Western blots with total cardiac tissue lysates (50 μg protein) from three MetS and three LD pigs using a specific antibody against GYS1 supported the RNA‐seq and protein mass spectroscopy observations (Figure 4c). However, the glycogenolysis‐involved muscle glycogen phosphorylase PYGM (mRNA, *p* = 0.052) and liver glycogen phosphorylase PYGL (protein *p* = 0.09, mRNA *p* = 0.12) are elevated in MetS versus LD. The glycogen phosphorylases' main function is to degrade glycogen, and their upregulation corresponds to the observed glycogen depletion in the MetS myocardium. Thus, the balance of GYS1 and glycogen phosphorylase expression and activity represent a powerful mechanism that ensures glycogen availability as a source of energy in the myocardium.

Neither glycogen synthase kinase 3 beta (GSK3ß), glycogen synthase kinase 3 alpha (GSK3α), (both key regulators of the function of glycogen synthase and thus cell glycogen levels), nor the phosphohexose isomerase (GDI), which catalyzes the reversible isomerization of GDP and GTP, exhibited diet‐related mRNA and protein variations in content. These enzymes served as internal controls for the observations made above (Figure 4b).

**FIGURE 5:**
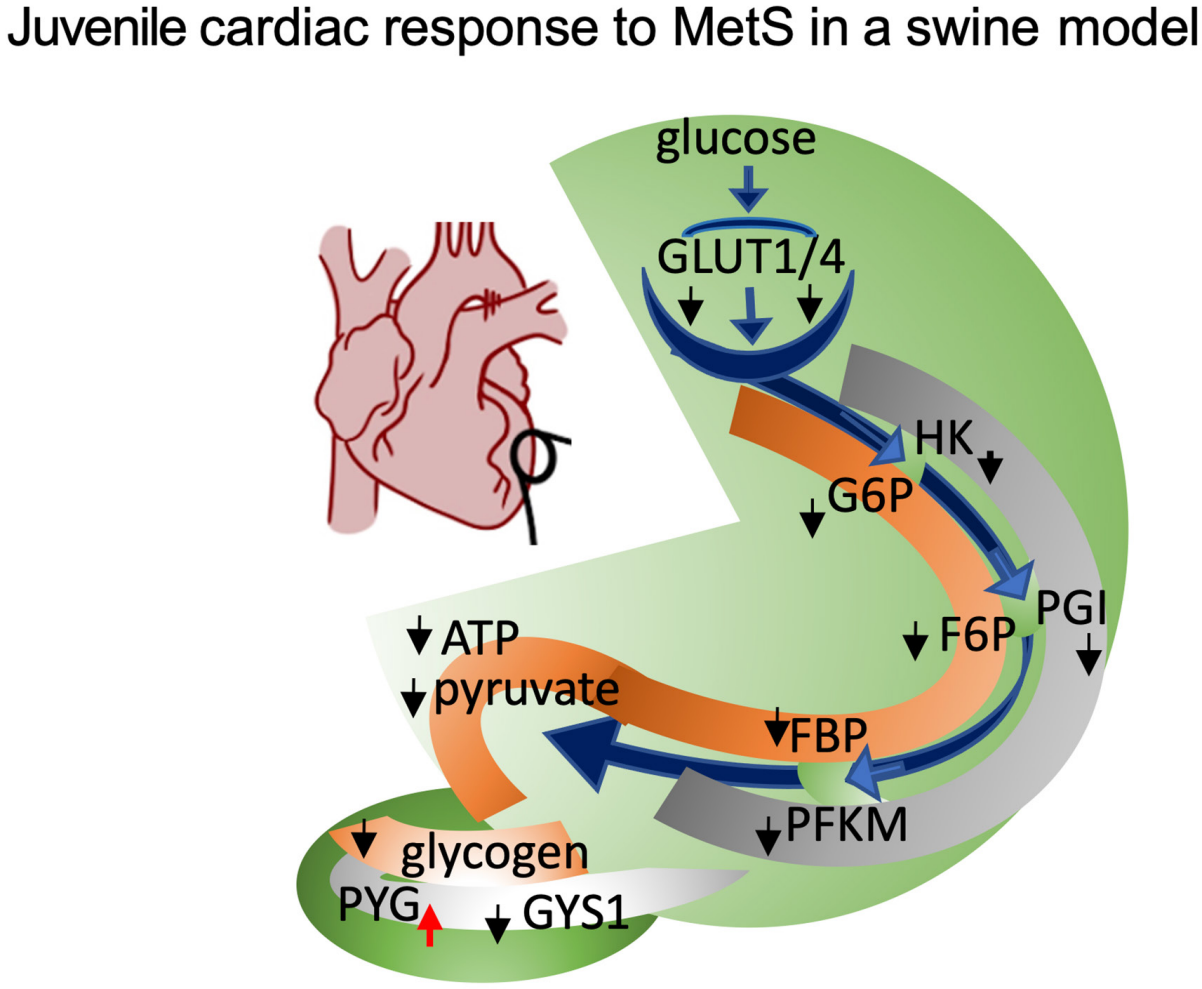
Graph showing the impact of MetS on glycolysis and glycogen in the juvenile myocardium. Glycolysis and glycogen‐related enzymes that are altered in MetS are highlighted in gray; orange – metabolic intermediates; black arrow denotes downregulation in MetS; red arrow denotes upregulation in MetS; the glycolysis pathway is shown in green and the glycogen is highlighted in dark green.

When taken together, these findings point to impaired glycolytic flux and loss of glycogen in the juvenile myocardium under conditions of hyperglycemia, obesity, hypertension, and ischemia which result in dysregulated glucose metabolism homeostasis and ATP availability (Figure 5).

## 4 DISCUSSION

Obesity is becoming more prevalent in children and adolescents, coinciding with rising MetS rates. Obesity in children is associated with abnormal glucose metabolism, dyslipidemia, inflammation, and compromised vascular function, all of which are components of MetS reproduced in our juvenile swine model. Given the role of MetS in metabolic changes the alteration in cardiac tissue metabolism in Mets has been an area of intense investigation. Previous studies have investigated various MetS‐related processes including abnormal gene expression, mitochondrial function, ion channel regulation, hemodynamics, and electrophysiology. Despite this research, the complexity of MetS has left a myriad of metabolic alterations that are not yet fully explored or even discovered. Few researchers have looked at energy substrate metabolism and the myocardial function as paradigm for the treatment of heart failure despite the fact that metabolism and myocardial function are inextricably linked.

To bridge gaps in our understanding of the myocardial response to MetS, and further unravel the molecular basis of predisposition to cardiovascular dysfunction in children, we adopted a multiomics approach that includes metabolomics, transcriptomics, and proteomics combined with physiologic data. By utilizing the computational NMF approach, we were able to integrate the various layers of information derived from the multiomics approach, resulting in identification of new key regulatory nodes in the control of the juvenile myocardial response to MetS.

In addition to the detrimental effects of the proinflammatory state associated with MetS, hyperglycemia also adversely affects myocardial glucose uptake. Our findings indicate that MetS animals have impaired myocardial glucose absorption; specifically, we noticed significantly lower protein levels of GLUT1 and GLUT4, reducing the transport of glucose across cellular plasma membranes. The low levels of mitochondrial HK1, which is required for the first mandatory step of glucose metabolism, resulted in low G6P availability in MetS swine. The role of G6P in glucose metabolism has been well‐established as the metabolic hub connecting glycolysis, glycogen synthesis, and the pentose phosphate pathway (PPP). G6P and its isomerization product, F6P, were both significantly reduced in the MetS heart. The decreased availability of F6P in MetS swine might reflect the limited availability of the glycolytic isomerase, PGI. In addition to its function as a glycolytic enzyme, PGI is a significant angiogenic factor that promotes endothelial cell motility (Fairbank et al., 2009). Of note, low PGI availability correlates with myocardial collateral deficiency in MetS and ischemia, which we previously reported in the adult pig myocardium (Karimi et al., 2020). Importantly, we observed no difference in PGI mRNA availability between MetS and LD swine, suggesting that this multifunctional and highly conserved enzyme is post‐translationally downregulated in the hyperglycemic juvenile myocardium. It is possible that low PGI and F6P are associated with myocardial MetS morbidity and may be of value as therapeutic targets.

Through a series of enzyme‐mediated glycolysis steps, F6P is further converted into FBP, pyruvate, and ATP. In MetS and ischemia, levels of FBP, pyruvate, and ATP were all low in the juvenile heart. Reduced F6P precursor availability, low PFKM enzyme availability, and mRNA expression are all possible contributing factors to lower FBP availability in MetS. Moreover, the phosphorylation reaction is slowed down even though ATP, the allosteric inhibitor of the PFKM‐catalyzed F6P phosphorylation to FBP, is not readily available in MetS.

The FBP insufficiency has a negative impact on glycolysis due to its dual roles as an allosteric activator of pyruvate kinase and as a precursor to glyceraldehyde‐3‐phosphate (G3P). It should also be noted that FBP alone has functions that are unrelated to the glycolysis pathway. It acts as an antioxidant by binding and sequestering soluble Fe(II) preventing its oxidation to insoluble Fe(III), which is a generator of reactive oxygen species via the Fenton reaction (Bajić et al., 2011). FBP has been shown to exert protective effects in a variety of ischemic injury models. FBP perfusion of isolated working rat hearts appears to directly affect myocardial hemodynamics, in contrast to glucose and fructose, which have no effect on any of the hemodynamic parameters (Cohen et al., 2006; Starnes et al., 1992; Veras et al., 2015). The exogenously delivered FBP may improve myocardial capacity in MetS and ischemia. The likely protective mechanism of FBP and the pharmacological profile of exogenous FBP in the myocardium in MetS and ischemia remains to be determined.

Metabolic syndrome consists of a cluster of metabolic risk factors, including insulin resistance, which increases the risk for CVD (Abel, 2005). The reduced abundance of key metabolites of the glycolysis pathway is proportional to insulin resistance in MetS. The terminal glycolysis product, pyruvate, was clearly diminished. Reduced levels of pyruvate and ATP in the MetS myocardium results in inadequate fueling of myocardial activities. Furthermore, as pyruvate in ischemia and MetS is low, compensatory glycogenesis in the MetS myocardium is unlikely to proceed (Gray et al., 2015). This observation is important as pyruvate is considered to be a metabolic protector of cardiac performance (Hermann et al., 1999; Karlstaedt et al., 2020; Schillinger et al., 2011). In addition to serving as an intermediary in a number of metabolic processes, pyruvate is a potent antioxidant that could strengthen cardiac oxidant defense (Mallet et al., 2002). As an adjunct inotropic agent, pyruvate could improve myocardial function in MetS by enhancing both systolic and diastolic myocardial function, thereby increasing ejection fraction without increasing heart rate (Hermann et al., 1999; Karlstaedt et al., 2020).

Alteration in metabolite and protein levels in the G6P‐pyruvate axis is a notable trait of the juvenile myocardium that may be particular to MetS and chronic ischemia. Beginning with the decreased availability of GLUT 1/4 and HK1, which catalyze the rate‐limiting and first necessary step of glucose metabolism, as well as the multifunctional PGI and PFKM, the juvenile myocardium's glycolysis appears to be dysregulated in MetS at the protein level, mRNA level, and availability in glycolysis metabolic intermediates.

The diminished glucose flux through glycolysis indirectly decreases another energy source for the heart, glycogen. Our findings suggest that chronic ischemia and MetS not only diminish glucose flux through glycolysis but also indirectly decrease the glycogen energy stores of the juvenile heart. Although glycogen is the primary cell deposit form for glucose, its true metabolic role in the myocardium is still being debated. Glycogen, along with PYGM, has recently been linked to myocardial contractile function (Testoni et al., 2017). We discovered a statistically significant decrease in glycogen availability in the myocardium of pigs with MetS and ischemia, which aligns with a decrease in intracellular glucose availability and oxygen supply.

Our mRNA data analysis revealed a MetS and ischemia‐related transcriptional signature, which may help to partially explain the loss of glycogen in the myocardium. GYS1 is vital for glycogen synthesis in the heart. This enzyme, however, was reduced significantly in the MetS myocardium, possibly reducing the ability for glycogen production. It is important to point out that the GYS1 regulator GSK3B is most likely not involved in GYS1 regulation and glycogen availability because its protein and mRNA levels in the MetS myocardium remain unchanged. Aside from the GYS1 limitation, the juvenile MetS myocardium expressed more PYGM and PYGL, which break down glycogen. The GYS1‐PYGM/L activity and expression balance may be a mechanism of control of glycogen availability. These findings are especially noteworthy because such an imbalance could contribute significantly to glycogen depletion, resulting in altered myocardial functionality. While other enzymes may play additional regulatory roles in certain contexts, our findings show that glycogen availability in the juvenile myocardium is may be controlled by a small number of pathways steps whose altered regulation underlies myocardial energy vulnerability in MetS.

We do not know if the myocardial response to MetS and oxygen deprivation differs by gender. Both male and female juveniles may have a similar glycolysis‐related response to ischemia alone, according to our unpublished findings. We will, however, be able to define the detailed metabolic gender‐specific energy response using our multiomic approach and the swine model.

The heart is able to utilize various sources of energy. Fatty acid oxidation is the primary energy source at rest, while glucose oxidation is utilized during increased effort and exertion (Taegtmeyer, 1994). Furthermore, it has been well‐established that cardiac tissue preferentially undergoes glycolysis in the setting of ischemia (Schirone et al., 2022). This metabolic flexibility serves as a protective mechanism that ensures the heart's functional continuity. However, our findings indicate that MetS and myocardial ischemia increases risk of cardiac dysfunction, which may be attributed to reduced availability and activity of substrates and enzymes required for glycolysis. This is further compounded by depletion of glycogen, a primary storage form of glucose in the myocardium, which plays a significant role in the heart's response to physiologic and pathophysiologic stress. to oxidize fat and carbohydrates, either simultaneously or vicariously.

### 4.1 Limitations

Our investigation was not without its limitations. We do not know if the myocardial response to MetS and oxygen deprivation differs by gender. Both male and female juveniles may have a similar glycolysis‐related response to ischemia alone, according to our unpublished findings. We will, however, be able to define the detailed metabolic gender‐specific energy response using our multiomic approach and the swine model. We were also limited by the use of an animal model itself. While a large animal model is a more accurate facsimile to human physiology than say mice, the expense and difficulty working with such a model inherently lowers the number of experimental animals that can be used and, therefore, reduces the power of the study. Furthermore, because of this model morphometric functional data, such as echocardiography, were not able to be obtained.

## 5 CONCLUSION

Our findings shed light on novel mechanisms by which diet‐induced MetS alters glucose metabolism at the level of metabolite availability and specific gene expression balance, emphasizing the critical role of metabolic reprogramming in cardiac adaptation to the plethora of risk factors in MetS and ischemia. The current data present a putative mechanism to add to the already well‐established increased cardiovascular risk factors that juvenile patients with MetS and known to have throughout their lives (Drozdz et al., 2021).

To this end, our findings support the concept of energy substrate metabolism and myocardial function as a paradigm for the treatment of heart failure.

## AUTHOR CONTRIBUTIONS

M.B. and D. H. conducted bioinformatics and metabolites and RNA extractions, disease‐related data evaluation and interpretation, and surgery; E.S. and A.S. conducted bioinformatics; S.S. and F.W.S. contributed physiological data, disease‐related data evaluation and interpretation, and surgery; B.S.A. NMF analysis; A.U. supervised the project, designed the experimental approach, conducted data analysis, wrote the manuscript, designed data presentation and graphics. All authors reviewed the manuscript.

## FUNDING INFORMATION

RO1 HL128831(AU), RO1 HL46716(FWS), NIH T32HL160517 to DH and MB; B.S.A. is supported by The Laboratory Directed Research and Development (LDRD) program of Los Alamos National Laboratory under project 20230067DR. Los Alamos National Laboratory is operated by Triad National Security, LLC, for the National Nuclear Security Administration of U.S. Department of Energy under Contract No. 89233218CNA000001.

## CONFLICT OF INTEREST STATEMENT

The authors declare that they have no competing interests.

## Supporting information


Figure S1

Figure S2

Figure S3

Table S1

## Data Availability

The RNA‐Seq data are available under GEO accession number PRJNA544355. The targeted polar metabolites distribution in MetS and LD tissue and blood are shown in the (Table S1). The proteomics LC/MS–MS data are available upon request.
