# Supplementary material for: Impaired cardiac glycolysis and glycogen depletion are linked to poor myocardial outcomes in juvenile male swine with metabolic syndrome and ischemia
Source: Physiol Rep. 2023 Aug 3;11(15):e15742. doi: 10.14814/phy2.15742 (PMC10400405; doi:10.14814/phy2.15742)
Supplement: Supplementary file 1 — Figure S1 [file PHY2-11-e15742-s002.pdf]

Figure S1. g:GOSt multiquery diagram of pathways with differential response to diet. GO:GOSt multiquery diagram summarizes results from RNA-seq. The Gene ontology molecular function data source (GO:MF) was used to identify pathways based on molecular function in the myocardial response to diet. The term name column shows the individual molecular functions; Term ID; p_{adj} (<0.05) –adjusted p-value for the association of the pathways with the differential response to diet. Selected pathways in the diagram are those with the highest significance (p_{adj} < $1e^{-3}$) in the corresponding data source. The bar diagrams on right show the negative logarithm of the p_{adj} value. The $-log_{10(padj)}$ axis is shown at the top of the bar diagrams and signifies the molecular functions with the strongest associations have smallest p_{adj} values and their negative logs are the greatest-bar color from dark green to yellow (16-0 $-log_{10(adj)}$).Some glucose-related molecular functions are highlighted in blue.

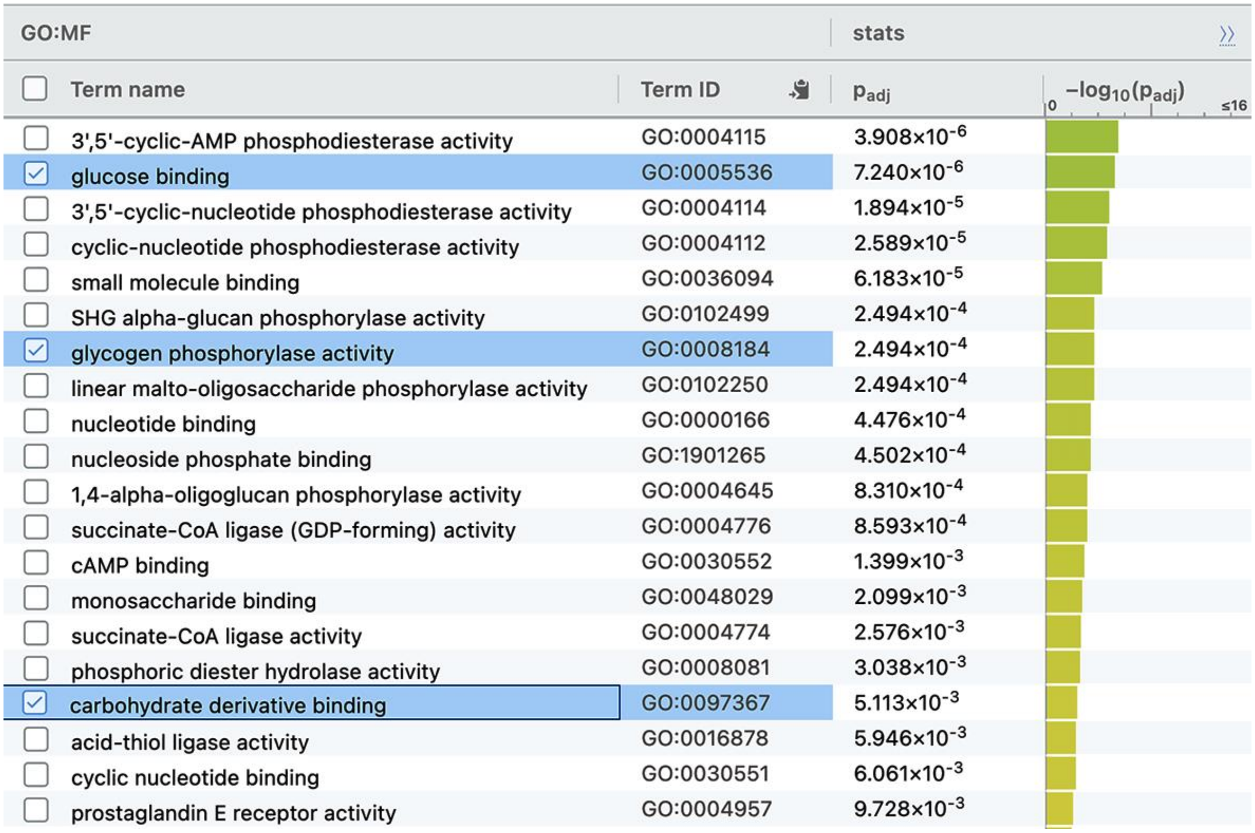

GO:MF		stats	
Term name	Term ID	p_{adj}	$-log_{10}(p_{adj})$ 0 … ≤16
3',5'-cyclic-AMP phosphodiesterase activity	GO:0004115	3.908×10^{-6}	
glucose binding	GO:0005536	7.240×10^{-6}	
3',5'-cyclic-nucleotide phosphodiesterase activity	GO:0004114	1.894×10^{-5}	
cyclic-nucleotide phosphodiesterase activity	GO:0004112	2.589×10^{-5}	
small molecule binding	GO:0036094	6.183×10^{-5}	
SHG alpha-glucan phosphorylase activity	GO:0102499	2.494×10^{-4}	
glycogen phosphorylase activity	GO:0008184	2.494×10^{-4}	
linear malto-oligosaccharide phosphorylase activity	GO:0102250	2.494×10^{-4}	
nucleotide binding	GO:0000166	4.476×10^{-4}	
nucleoside phosphate binding	GO:1901265	4.502×10^{-4}	
1,4-alpha-oligoglucan phosphorylase activity	GO:0004645	8.310×10^{-4}	
succinate-CoA ligase (GDP-forming) activity	GO:0004776	8.593×10^{-4}	
cAMP binding	GO:0030552	1.399×10^{-3}	
monosaccharide binding	GO:0048029	2.099×10^{-3}	
succinate-CoA ligase activity	GO:0004774	2.576×10^{-3}	
phosphoric diester hydrolase activity	GO:0008081	3.038×10^{-3}	
carbohydrate derivative binding	GO:0097367	5.113×10^{-3}	
acid-thiol ligase activity	GO:0016878	5.946×10^{-3}	
cyclic nucleotide binding	GO:0030551	6.061×10^{-3}	
prostaglandin E receptor activity	GO:0004957	9.728×10^{-3}	
