# Supplementary material for: Impaired cardiac glycolysis and glycogen depletion are linked to poor myocardial outcomes in juvenile male swine with metabolic syndrome and ischemia
Source: Physiol Rep. 2023 Aug 3;11(15):e15742. doi: 10.14814/phy2.15742 (PMC10400405; doi:10.14814/phy2.15742)

Figure S2. (related to Fig. 3A,B Fig.4B) Control genes and metabolites that do not have a significant change in LD and MetS. RNA-seq, proteomics, and targeted polar metabolites LC/MS-MS were applied to identify and compare contents of gene products and metabolites. Myocardial mRNA, proteomic, and targeted polar metabolites libraries from LD (n=4) pigs and MetS (n=4) pigs are compared for relative content of genes and metabolites. Parallel blood samples LD (n=4) and MetS (n=4) are used to perform metabolomic analyses. (**A**) Genes that do not change significantly at the mRNA and protein level in response to diet. The abundantly expressed genes are compared via mRNA levels: METTL1, P2RY6, P2RY12. Desmin is compared at mRNA and at mass spectroscopy derived protein level. Values are in relative units (R.U.); as shown on the verticals; means +/-SD, P<0.05. (**B**) the polar metabolite, N-Acetyl-L-alanine, does not respond to diet in tissue. **(c)** Metabolites that are highly abundant in the blood vs tissue in both LD and MetS: D-glyceraldehdye-3-phosphate and ascorbic acid. **(d)** The D-sedoheptulose-1-7-phosphate is nearly identical in tissue and blood, with no reaction to diet. The genes and metabolites are shown at the top of the diagrams. The color identity of the bars is shown at the bottom. Values are in relative units; all bar diagrams are mean +/-SD, P<0.05. 200 µg of protein per tissue and blood sample was subjected to proteomic and metabolomic LC/MS-MS. RNA-seq is performed with 50 mg total RNA per tissue sample.

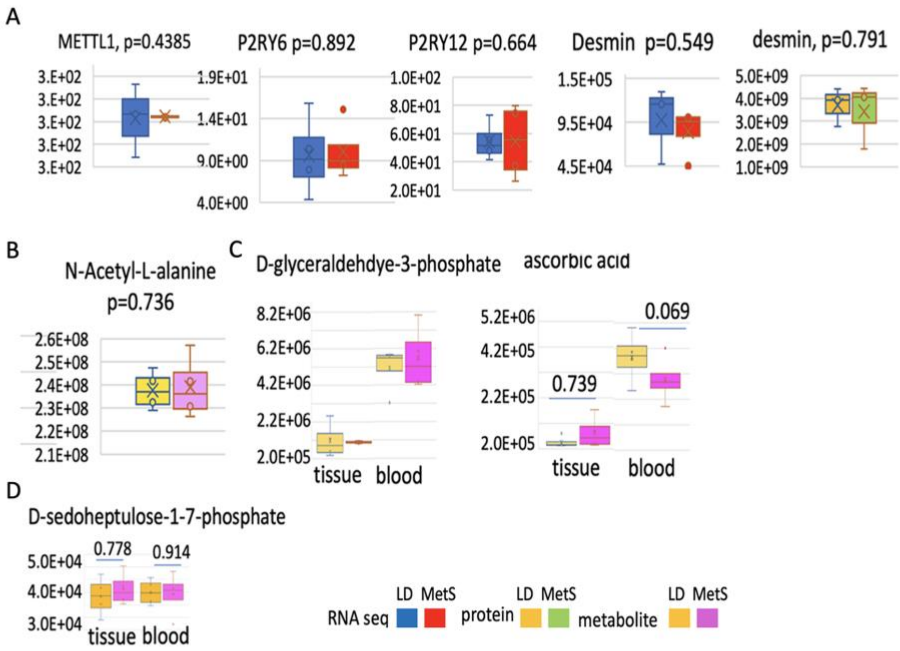

Supplement: Supplementary file 2 — Figure S2 [file PHY2-11-e15742-s001.pdf]
