# Supplementary material for: Impaired cardiac glycolysis and glycogen depletion are linked to poor myocardial outcomes in juvenile male swine with metabolic syndrome and ischemia
Source: Physiol Rep. 2023 Aug 3;11(15):e15742. doi: 10.14814/phy2.15742 (PMC10400405; doi:10.14814/phy2.15742)

Figure S3 (related to Fig. 4c). Ponceau staining of the membrane confirmed equal lysate loading and transfer

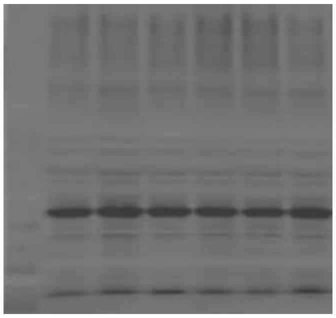

Supplement: Supplementary file 3 — Figure S3 [file PHY2-11-e15742-s004.pdf]
