# Supplementary figures and images for: Impaired cardiac glycolysis and glycogen depletion are linked to poor myocardial outcomes in juvenile male swine with metabolic syndrome and ischemia
Source: Physiol Rep. 2023 Aug 3;11(15):e15742. doi: 10.14814/phy2.15742 (PMC10400405; doi:10.14814/phy2.15742)

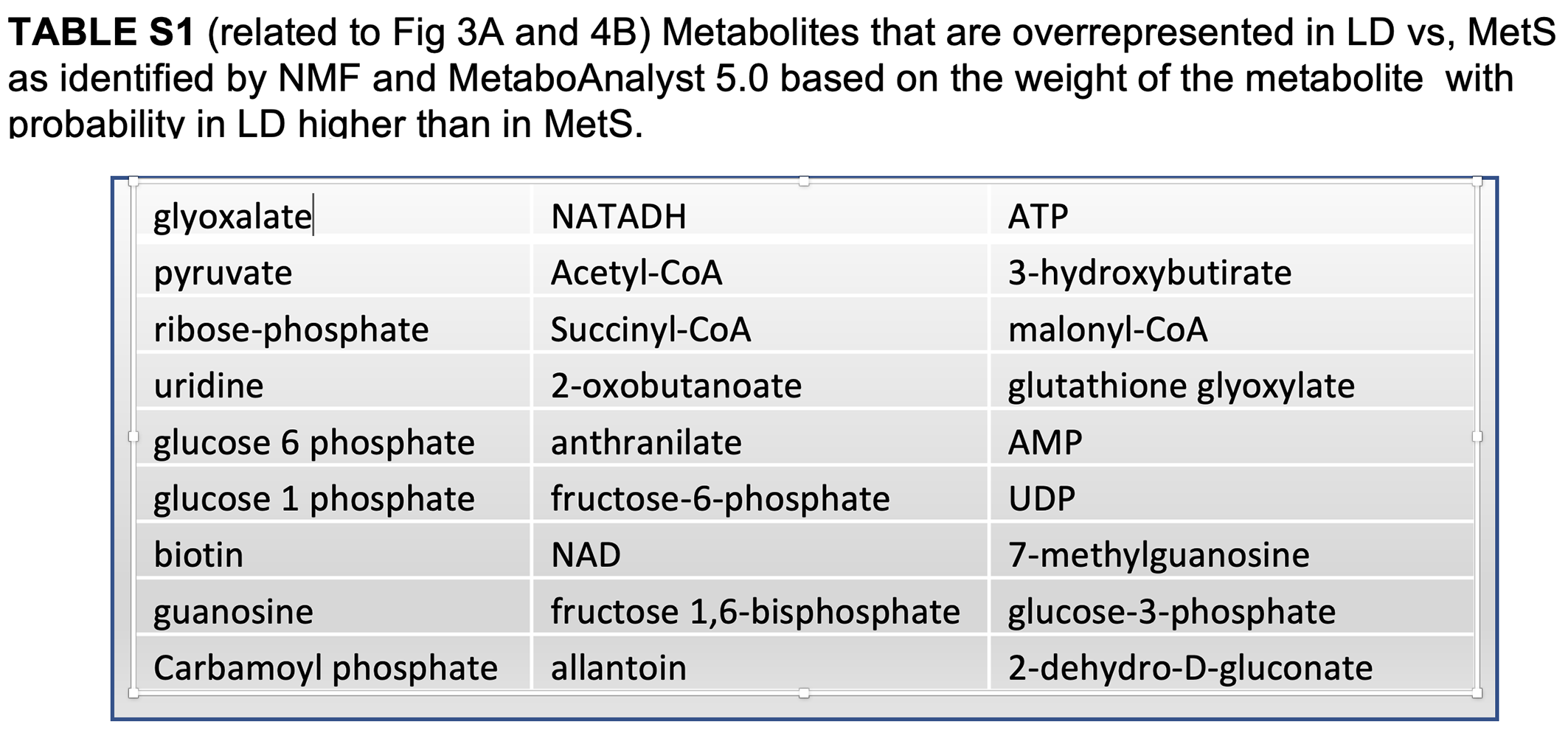

Supplement: Supplementary file 4 — Table S1 [file PHY2-11-e15742-s003.docx]
